# COVID-19 and violence: a research call to action

**DOI:** 10.1186/s12905-020-01115-1

**Published:** 2020-11-10

**Authors:** Dabney P. Evans

**Affiliations:** Hubert Department of Global Health, Emory University, 1518 Clifton Rd, NE, Atlanta, GA 30322 USA

**Keywords:** COVID-19, Violence against women, Intimate partner violence, Pandemic, Emergencies, Research

## Abstract

COVID-19 related guidelines and movement restrictions are designed to protect the public’s health and reduce disease transmission; yet, COVID-19 related restrictions on movement including social distancing, isolation, quarantine, and shelter-in-place orders have an unknown effect on violence and abuse within relationships. As the pandemic has progressed, many have justifiably speculated that such restrictions may pose a danger to the safety and well-being of people experiencing such violence. Early in the pandemic, countries hard hit by COVID-19 began raising the alarm bell about the impacts of the disease on IPV occurrence. Police in China report that 90% of the causes of recent IPV cases could be attributed to the COVID-19 epidemic. Rising fears and anxiety about prolonged movement restrictions, increased economic strain and diminished health care capacity to support survivors are among the potential reasons for such dramatic effects. Under normal circumstances: low income, unemployment, economic stress, depression, emotional insecurity and social isolation are all risk factors for using violence against partners. Many of these factors may worsen in the context of COVID-19. Despite the urgency in addressing COVID-19, existing health concerns like Intimate Partner Violence (IPV) persist—and may well be worsened by the virus. We simply do not yet know the effects of COVID-19 on violence, nor do we know which interventions work best to prevent and respond to it within the context of the pandemic. The vast majority of information available about IPV and violence during the pandemic has been based on anecdotal reports. The call to action for the research community is clear. We must systematically measure the effects of COVID-19 and movement related restrictions on violence. As always when researching violence, serious consideration must be given to ethics and safety. Violence researchers must mobilize to investigate the impacts of COVID-19 on violence and human health.

COVID-19 related guidelines and movement restrictions are designed to protect the public’s health and reduce disease transmission; yet, COVID-19 related restrictions on movement including social distancing, isolation, quarantine, and shelter-in-place orders have an unknown effect on violence and abuse within relationships. As the pandemic has progressed, many have justifiably speculated that such restrictions may pose a danger to the safety and well-being of people experiencing such violence. Intimate Partner Violence (IPV) includes physical, sexual, and psychological abuse by a past or current intimate partner [1]. IPV is incredibly common; for example, in the United States, nearly 20 people every minute are physically abused by an intimate partner [2]. While physical violence is the most obvious form of abuse, IPV can also include less identifiable forms of abuse that either center around intimidation and control or are an exacerbation of those who use physicality when they “fight” [1]. Such controlling behaviors are likely to be exacerbated during periods of movement restriction with the pandemic being used as a justification for increased control.

Early in the pandemic, countries hard hit by COVID-19 began raising the alarm bell about the impacts of the disease on IPV occurrence. France saw a 36% increase in the number of reported IPV cases and one city in China reported a two-fold increase in IPV cases in one month following movement restrictions [3, 4]. Police in China reported that 90% of the causes of recent IPV cases could be attributed to the COVID-19 epidemic [4]. Rising fears and anxiety about prolonged movement restrictions, increased economic strain and diminished health care capacity to support survivors are among the potential reasons for such dramatic effects.

Under normal circumstances: low income, unemployment, economic stress, depression, emotional insecurity and social isolation are all risk factors for using violence against partners. Many of these factors may worsen in the context of COVID-19 [5, 6]. Notably, increased social isolation is an actively—and justifiably—promoted tactic to prevent disease transmission [7]. At the same time, isolation is sometimes used by those who use violence against partners to keep family problems secret or to exert power and control [1]. It also means that women who are experiencing violence have less chance to reach out to co-workers or extended family members as well as to other helping resources when they have to stay home [8]. Yet, those isolated at home still have options, albeit more limited than usual. Those in violent relationships may feel that leaving during a pandemic is unfeasible or undesirable. For those people, safety planning apps like myPlan—which is available app free in all app stores—can be used to take a homicide risk assessment (the Danger Assessment) and create a personalized safety plan [9–11]. Family members and friends can also use myPlan on behalf of people they are worried about.

Despite the urgency in addressing COVID-19, existing health concerns like IPV persist—and may well be worsened by the virus [12]. Immediate interventions such as the use of code words at pharmacies, and exemptions on movement restrictions for IPV survivors have reportedly shown promise in responding to survivors immediate needs [13]. Such interventions may also assist in repurposing industries hit hard by the economic effects of COVID-19. For example, the provisions of hotel rooms for survivors by the French government is at once serving survivors immediate needs and bolstering support for the weakened hospitality industry; similar efforts have been supported by hotel associations in the US [3, 14].

However promising such programs may be a glaring gap remains: we simply do not know the effects of COVID-19 on violence, nor do we know which interventions work best to prevent and respond to it within the context of the pandemic. The vast majority of information available about IPV and violence during the pandemic has been based on anecdotal reports [3, 4, 8, 13]. The call to action for the research community is clear. We must systematically measure the effects of COVID-19 and movement related restrictions on violence. This includes not only incidence of IPV—and other forms of gender-based violence—but also the risk factors for violence perpetration, victimization and the socio-contextual determinants influencing the occurrence of violence during this time. As always when researching violence, serious consideration must be given to ethics and safety [15–17]. Fortunately, several UN agencies have already offered resources and guidance on how to safely and ethically conduct research during this period [18–20].

Admittedly, the typical timeline for research is dramatically slower than social phenomena even in the absence of a pandemic. A lack of data should not prevent the development and implementation of new and adapted programs to respond to violence during this period. After all, we already know how prevalent it is; violence itself is a pandemic [21–23]. Scientists focused directly on addressing COVID-19 have coalesced to address the need for testing, treatments and a vaccine [24]. So too must violence researchers mobilize to investigate the impacts of COVID-19 on violence and human health.

## Data Availability

Data sharing not applicable to this article as no datasets were generated or analyzed during the current study.

## Abbreviations

COVID-19: Corona virus disease 2019
IPV: Intimate partner violence
UN: United Nations
US: United States
